# Metabolic Traits of Bovine Shiga Toxin-Producing *Escherichia coli* (STEC) Strains with Different Colonization Properties

**DOI:** 10.3390/toxins12060414

**Published:** 2020-06-22

**Authors:** Stefanie A. Barth, Michael Weber, Katharina Schaufler, Christian Berens, Lutz Geue, Christian Menge

**Affiliations:** 1 Friedrich-Loeffler-Institut/Federal Research Institute for Animal Health, Institute of Molecular Pathogenesis, Naumburger Str. 96a, 07743 Jena, Germany; Michael.Weber@fli.de (M.W.); Christian.Berens@fli.de (C.B.); lutz.geue@fli.de (L.G.); Christian.Menge@fli.de (C.M.); 2 Free University Berlin, Institute of Microbiology and Epizootics, Robert-von-Ostertag-Str. 7-13, 14163 Berlin, Germany; Katharina.Schaufler@uni-greifswald.de; 3 University of Greifswald, Pharmaceutical Microbiology, Friedrich-Ludwig-Jahn-Str. 17, 17489 Greifswald, Germany

**Keywords:** Shiga toxin-producing *Escherichia coli*, STEC, colonization type, Omnilog, metabolic activity, biofilm, acid resistance, bovine, reservoir

## Abstract

Cattle harbor Shiga toxin-producing *Escherichia coli* (STEC) in their intestinal tract, thereby providing these microorganisms with an ecological niche, but without this colonization leading to any clinical signs. In a preceding study, genotypic characterization of bovine STEC isolates unveiled that their ability to colonize cattle persistently (STEC^per^) or only sporadically (STEC^spo^) is more closely associated with the overall composition of the accessory rather than the core genome. However, the colonization pattern could not be unequivocally linked to the possession of classical virulence genes. This study aimed at assessing, therefore, if the presence of certain phenotypic traits in the strains determines their colonization pattern and if these can be traced back to distinctive genetic features. STEC^spo^ strains produced significantly more biofilm than STEC^per^ when incubated at lower temperatures. Key substrates, the metabolism of which showed a significant association with colonization type, were glyoxylic acid and L-rhamnose, which were utilized by STEC^spo^, but not or only by some STEC^per^. Genomic sequences of the respective *glc* and *rha* operons contained mutations and frameshifts in uptake and/or regulatory genes, particularly in STEC^per^. These findings suggest that STEC^spo^ conserved features leveraging survival in the environment, whereas the acquisition of a persistent colonization phenotype in the cattle reservoir was accompanied by the loss of metabolic properties and genomic mutations in the underlying genetic pathways.

## 1. Introduction

Enterohemorrhagic *Escherichia coli* (EHEC), a subgroup of Shiga toxin-producing *E. coli* (STEC), pose a risk to humans, especially infants and children, by causing diseases ranging from mild diarrhea to life-threatening hemorrhagic uremic syndrome (HUS). The STEC pathovar consists of a plethora of different strains sharing a single property, the eponymous, highly toxic Shiga toxin (Stx). This protein exists in two serologically differentiable forms, Stx1 and Stx2, which can be further divided into subtypes (Stx1a, Stx1c, Stx1d, and Stx2a through Stx2h) [1,2]. Besides Stx, STEC strains may possess additional virulence traits such as adhesion factors, protein secretion systems or additional toxins, partially encoded on mobile genetic elements, such as plasmids or pathogenicity islands. The resulting very high genomic flexibility of this pathovar is reflected by the fact that strains from more than 400 different *E. coli* serotypes are known to encode Stx. Yet, only very few of these, including those possessing O-antigens O26, O45, O103, O111, O121, O145, and O157 [3], are responsible for the majority of the human infections.

Cattle harbor STEC in their intestinal tract without displaying any clinical symptoms, thereby providing an ecological niche for the bacteria. Numerous attempts have been undertaken to subdivide the many different STEC strains that are shed by cattle in order to predict a given strain’s degree of threat to human health. Various levels of host adaptation could be traced back to certain patterns of virulence genes and their expression levels. EHEC O157:H7 strains, e.g., were found to express *iha*, *espA*, *rfbE*, and *ehxA* to different extents upon natural infections of humans and cattle [4]. Spontaneous Stx production is higher in HUS-associated EHEC clones than in bovine STEC isolates, and Stx1 production is induced more strongly by iron deprivation in vitro in the former [5]. A lower capacity to produce Stx2 in bovine STEC correlates with the presence of the Q_21_ allele of the late antiterminator Q upstream of *stx* in the genome of *stx*-converting prophages, whereas strongly inducible Stx production seems to be linked to the Q_933_ allele [6]. Indeed, a support vector machine analysis of bovine *E. coli* O157 isolate sequences, by comparison with sequences from human isolates, identified cattle strains more likely to be a serious threat to human health [7]. Distinction was possible despite the fact that the majority of the isolates considered were members of previously defined pathogenic lineages and encoded key virulence factors. The major differences between human and bovine *E. coli* O157 isolates were the relative abundances of predicted prophage proteins. However, the predictive value for human pathogenicity of such analyses was severely questioned by the appearance of unusual EHEC strains possessing a blended virulence profile combining genetic patterns of EHEC and human-adapted enteroaggregative *E. coli* (EAEC), rarely detected in animal hosts before [8,9]. Although the O104:H4 EHEC/EAEC hybrid strain, having caused the 2011 German outbreak, appears to be preferably adapted to humans, the strain’s ability to colonize the intestinal epithelial cells of humans and cattle [10] indicates that even EHEC strains with an unusual genotype can colonize other reservoir hosts. Indeed, the outbreak strain colonizes calves under experimental conditions [11], its genetic markers are present in the cattle population [12], and the strain has been grouped in the midst of bovine commensal strains in a recent comprehensive genome analysis unveiling the evolutionary sources of the emergence of human intestinal pathogenic strains [13].

Although many human EHEC infections originate from ruminants as a direct or an indirect source of infection, the molecular basis of the complex shedding and transmission dynamics of the plethora of STEC strains in animal populations is poorly understood. Cumulative evidence exists that STEC strains isolated from cattle can be subdivided into subsets of strains based on their colonization pattern in the reservoir host. Stx1 and Stx2 have been shown to suppress host-adaptive immune responses [14,15,16] affecting STEC colonization and shedding [17]. Certain Stx subtypes, in particular Stx2a, are epidemiologically associated with increased excretion levels of *E. coli* O157 from cattle, also known as super-shedding [18,19,20]. Stx2a increases the efficiency of *E. coli* O157 transmission between animals, presumably because it is more rapidly produced than Stx2c and restricts cellular proliferation of bovine epithelial cells [21]. A previous longitudinal study by our group identified STEC strains belonging to distinct serotypes that were repeatedly isolated from cattle feces over a period of several months, while other strains were isolated only at single or a few time points [22]. This observation allowed us to group these strains into either persistent (STEC^per^) or sporadic (STEC^spo^) colonizers of the bovine intestine. Interestingly, the majority of bovine STEC^per^ strains belonged to other serotypes (O156, O165) than those commonly linked to human infections [22,23,24], although O26:H11 were also found to be STEC^per^ [25]. An initial genetic analysis of STEC^per^ and STEC^spo^ strains revealed that the capacity to colonize cattle correlates with the accessory genome and the presence of virulence-associated genes (VAG) and not with the core genome, represented by multilocus sequence typing (MLST) [26]. However, no single genes or gene clusters could be directly associated with the colonization type despite the fact that several genes displayed a significantly higher prevalence in one of the groups. The genes *stx1*, *eae*, *efa*-1, *lpfA* or several type III secretion system-associated genes are more often found in STEC^per^, while *stx2*, *toxB* or *cdt* are more often present in STEC^spo^.

The subgroup of STEC^per^ forms a resilient VAG gene pool within the mixing vessel of the ruminant’s intestine that may build the basis for the evolution of new zoonotic strains. Studying the genomic content of strains provides information on a strain’s potential, but it is mandatory to evaluate which genetic properties are actually translated into phenotypes if we aim at a better understanding of the molecular basis behind the varying lifestyles that STEC strains realize in cattle populations. This includes properties that enable strains to stand out, perhaps as a small proportion of the entire *E. coli* population, by being able to colonize or survive in particular ecological niches. This might occur through metabolizing special nutrients or macroelements such as a carbon, nitrate, phosphorus or sulfate source, through pronounced acid resistance, or through occupying or even contributing to specific niches, e.g., by biofilm formation. Therefore, we assessed phenotypic properties of 28 STEC strains representing different colonization types, VAG-patterns, and MLSTs, selected as being representative of a set of 178 Illumina whole genome-sequenced STEC strains [26].

## 2. Results

### 2.1. Acid Resistance

The ability of the STEC strains to survive at low pH values was tested by incubation of approx. 7.3 log10 colony-forming units (cfu) per mL of each strain in LB broth adjusted to pH 1.5, pH 2.5 and, as control, pH 7.8. Surviving cells were quantified by cfu counts. These showed no general significant differences between the STEC^per^ and STEC^spo^ isolates (*p* > 0.05; Table 1), but rather between serotypes. The O156/O182 and O157 STEC strains were highly susceptible to acid stress as the incubation at pH 2.5 strikingly reduced the cfu, and only one strain survived at pH 1.5. In contrast, O26 STEC strains were highly resistant against acidic broth as all four strains withstood exposure to pH 1.5.

### 2.2. Biofilm, Curli, and Cellulose Production

**Figure 1:**
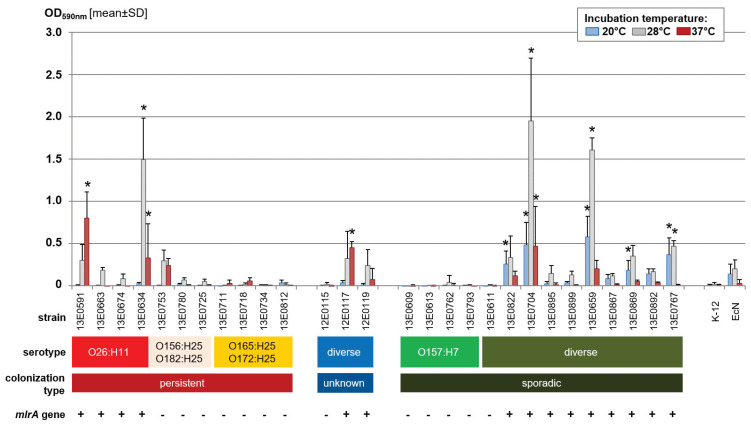
Biofilm production in polystyrene plates after incubation of the STEC strains at different temperatures in salt-free lysogeny broth. Results of the crystal violet assay are shown as the mean + standard deviation [SD] of four biological replicates. Significant differences in mean values relative to the negative control strain *Escherichia coli* K-12 are indicated by an asterisk (*p* < 0.05, Dunnett-T). The completeness of the *mlrA* gene is displayed by ‘+’ (complete, 243 amino acids) or ‘−’ (truncated, 215 amino acids) for each strain.

Analysis of STEC strains in the crystal violet plate assay yielded a broad variety of biofilm formation ability (Figure 1). While some strains markedly produced biofilm, even exceeding that of the positive control *E. coli* Nissle 1917 (e.g., strains 13E0634, 31E0704, and 13E0659), several strains produced no measurable biofilm (e.g., strains 12E0115, 13E0609, and 13E0611). Interestingly, strains defined as sporadically colonizing STEC produced significantly more biofilm on average when incubated at lower temperatures (20 °C) than STEC^per^ (*p* = 0.016). In contrast, STEC^per^ preferentially produced biofilm at 37 °C. O157-STEC strains displayed no or only very little biofilm formation, independent of the incubation temperature.

A search for biofilm-related genes (*bcs* genes, *csg* genes, *adrA*, *rpoS*, *mlrA*) in the WGS data of the strains showed only a correlation between the presence and integrity of *mlrA* in a given strain and its relative ability to form a biofilm (Figure 1).

**Figure 2:**
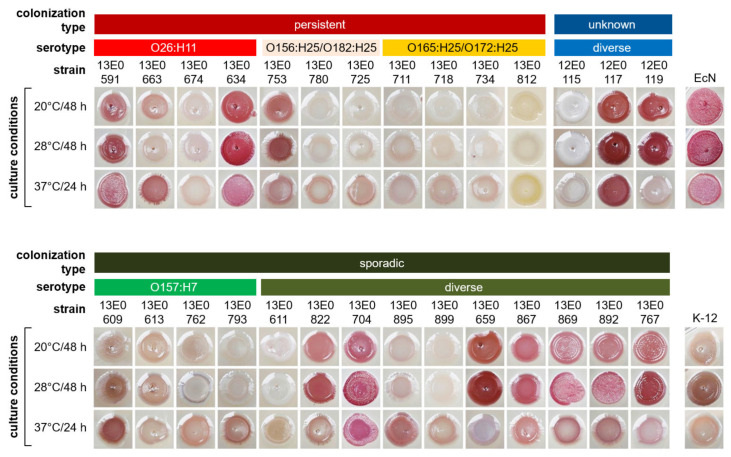
Curli expression of STEC strains after growth on Congo red agar. The plates were incubated at different temperatures for 24–48 h. After incubation, the colony morphology was macroscopically assessed and documented by photography.

These results were confirmed by incubation of the strains on Congo red agar (Figure 2). Here, strains with high-level biofilm formation in the crystal violet plate assay (e.g., 13E0591, 13E0634, 13E0704, and 13E0659) showed the production of curli (brown), cellulose (pink) or both (rdar phenotype) at the respective incubation temperature. Analyzing STEC^per^, especially O26 strains, produced biofilm at 37 °C as shown with the rdar phenotype. In STEC^spo^, the rdar phenotype was predominantly found after incubation at 20 or 28 °C. Also, in this assay, the O157 STEC^spo^ remained white, indicating no curli or cellulose production, or only exhibited a light brownish color after incubation at 37 °C.

### 2.3. Metabolic Activity of the Strains and Classification According to Their Colonization Type

Using the Omnilog^®^ phenotype microarray, the strains were tested for their utilization of 190 individual C-, 95 N-, 59 P-, and 35 S-sources during a 48 h incubation period at 37 °C. Inspection of the metabolic activity of the strains on each plate revealed that three strains belonging to the O165/O172 STEC cluster (strains 13E0711, 13E0718, and 13E0812) utilized fewer substrates than the other 25 strains (Figure S1). Interestingly, the fourth strain belonging to this cluster (O165:H25, 13E0734) grew in the presence of nearly half of the substrates and showed a mean metabolic activity comparable to many other strains. To analyze the metabolic patterns, we excluded the three poorly-metabolizing strains and analyzed the 25 remaining strains. In order to take the overall performance as well as the kinetics of substrate utilization into account, an area-under-the-growth curve approach (AUC) was applied, and the analysis was based on an AUC cutoff value of 500 (Table S1). Out of 379 substrates tested, 144 substrates were metabolized by 23 or more strains and 100 substrates were metabolized by no more than three strains. The remaining 135 substrates were metabolized to different extents, including 61 C-sources, 36 N-sources, 11 P-sources, and 27 S-sources, and were included in the further analysis.

**Figure 3:**
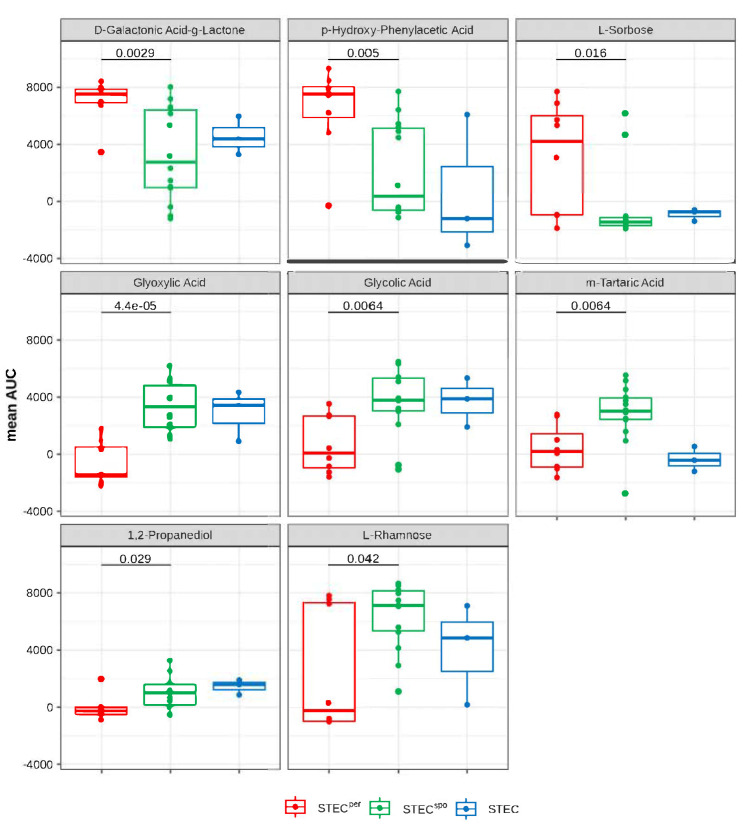
C-Substrates with significant differences in the metabolic activity between STEC strains able to colonize cattle persistently (STEC^per^) and only sporadically (STEC^spo^). Shown are the area-under-the-growth curve (AUC) values of each group as box-whisker plots of the discriminatory carbohydrate substrates with significant (*p* < 0.05) differences in their mean AUC values between STEC^per^ (red) and STEC^spo^ (green). The blue color depicts STEC with an unknown colonization type included for comparison.

To identify substrates that might discriminate between STEC^per^ and STEC^spo^, the mean AUC of each variably metabolized substrate was analyzed for significant differences between the two groups by two-sample Wilcoxon tests. As a result, the oxidization of eight C- and of seven S-sources differed significantly between the groups (Figure 3 and Figure S2). Values measured for the S-sources were very low and, although their mean AUC values differed significantly, the value ranges of both groups overlapped notably (Figure S2). Data for the metabolism of S-sources were therefore not analyzed further. In contrast, the value ranges of the metabolic activities determined for all eight C-sources differed significantly between the STEC^per^ and STEC^spo^ groups (Figure 3).

Only three C-substrates yielded a significantly higher mean AUC value in the STEC^per^ group (D-galactonic acid-γ-lactone [PM1_C02], p-hydroxy-phenylacetic acid [4-HPAA, PM1_H02], and L-sorbose [PM2A_D04]), while the remaining five C-substrates were significantly better metabolized by STEC^spo^ strains (glyoxylic acid [PM1_F10], glycolic acid [PM1_F09], tartaric acid [PM1_E02], 1,2-propanediol [PM1_D04], and L-rhamnose [PM1_C06]). Interestingly, three of the latter substrates (glyoxylic acid, glycolic acid, and tartaric acid) can be allocated to a single metabolic pathway, namely, glyoxylate metabolism.

**Figure 4:**
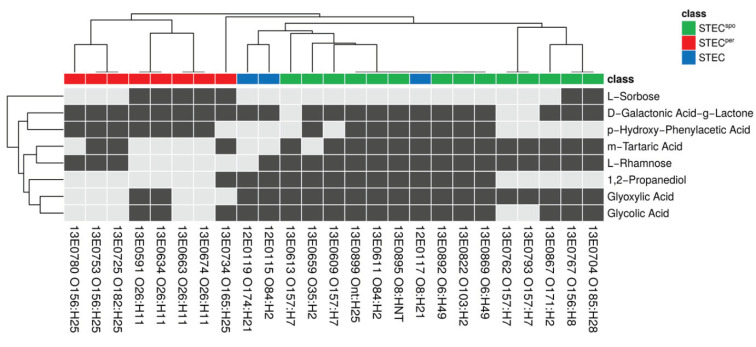
Individual profiles of C-substrates with significantly different metabolic activity between STEC^per^ and STEC^spo^ for each strain. Visualization of the binary substance matrix as a heatmap. The heatmap displays positive (black)/negative (grey) values for 25 columns (strains) and eight rows (discriminatory substrates). The annotation column “class” indicates the respective strain’s linked colonization type STEC^per^ (red) and STEC^spo^ (green). Rows and columns are ordered according to agglomerative clustering (average linkage).

Substrates with significant discriminating power in the statistical tests were further investigated by classification analysis. In a first step, we transformed the normalized AUC growth values according to the cutoff value of 500 into binary values (“positive” versus “negative”). This step resulted in a binary data matrix, which served as input for all following procedures. The matrix consisted of eight columns (discriminatory C-substrates) and 25 rows (STEC strains) and is visualized by a binary heatmap (Figure 4). Agglomerative clustering of the rows illustrated a clear separation of persistent and sporadic colonizers in the row dendrogram.

**Figure 5:**
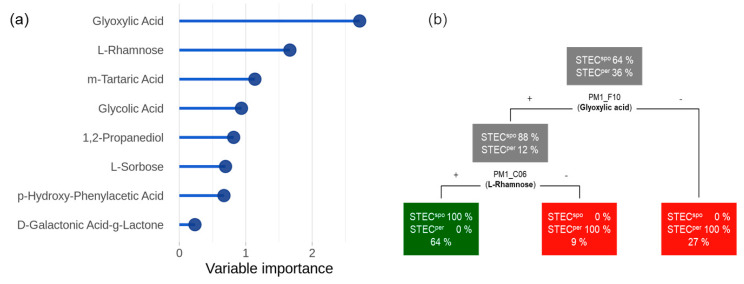
Feature importance and decision tree. Results of the classification analysis are displayed in a feature importance plot (**a**) and a summary decision tree (**b**). In (**a**), features are ordered with respect to their individual impact on classification accuracy. The decision tree in (**b**) is applied by top-down decisions that classify strains into colonization type based on the features glyoxylic acid and L-rhamnose.

However, a perfect discrimination between the colonization classes was not possible using just a single feature. Therefore, we conducted a multivariate classification with the objective to (1) identify relevant discriminatory substrates and (2) quantify their capability to predict the colonization type correctly. Using the random forest method, a ranked feature list ordered by variable importance was generated (Figure 5a). The most important feature was glyoxylic acid, followed by L-rhamnose and m-tartaric acid. A random forest classifier trained on the top feature (glyoxylic acid) resulted in a receiver operating characteristic (ROC) AUC value of 0.81 (95% CI: 0.77 to 0.84). Adding L-rhamnose to the training matrix increased the classification power up to 1.00 (95% CI: 0.99 to 1.00). Therefore, we considered glyoxylic acid and L-rhamnose as a minimal set in order to permit reliable predictions concerning the colonization type of an STEC strain. To analyze the decision process, we trained a single decision tree on the entire dataset (Figure 5b). This tree suggests that a strain is classified as STEC^per^ if it is either unable to metabolize glyoxylic acid or is capable of glyoxylic acid utilization but negative for L-rhamnose. Conversely, STEC^spo^ are strains which are positive for both glyoxylic acid and L-rhamnose metabolizing capability.

To verify the results obtained with the discriminatory substrates glyoxylic acid and L-rhamnose in the Omnilog^®^ system, we tested all 28 representative strains in M9 minimal medium supplemented with either of the substrates. The growth kinetics were measured for 24 h, and the AUC was calculated for each STEC strain. For direct comparison, we used the AUC of the Omnilog^®^ experiments also including only the first 24 h of the incubation. Resulting Pearson’s correlation coefficients showed that the results of both test systems corresponded well (Figure S3), as the mean AUC values of both test systems correlated at *r* = 0.822 (y = 0.1547x − 385.47) using glyoxylic acid as the C source and at *r* = 0.802 (y = 0.2475x − 267.77) when L-rhamnose was present.

### 2.4. Genetic Basis for Key Metabolic Reactions

As glyoxylic acid and L-rhamnose were identified to be key substrates for the classification of STEC^per^ and STEC^spo^, we checked the genetic organization of the strains with respect to the enzymes involved in the respective metabolic pathways.

**Figure 6:**
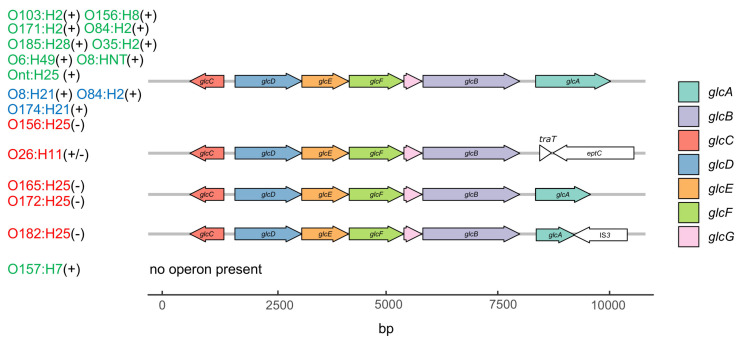
Gene map of different *glc* operons in bovine STEC. The transcription of the *glc* operon is regulated by integration host factor (IHF) and GlcC, the latter itself being modulated by the anoxic redox control regulators ArcA-P. The *glc* operon encodes subunits of glycolate oxidase (GlcD, GlcE, GlcF), malate synthetase (GlcB), and glycolate permease (GlcA). The function of GlcG is not clear [27]. Serotypes in red represent STEC^per^, in green STEC^spo^, in blue STEC with unknown colonization type; their respective glyoxylic acid utilizing phenotype is shown in parentheses (−, negative; +, positive). Strains included in this study and belonging to the same serotype did not differ in their *glc* operon composition and are therefore not shown individually in the figure.

**Figure 7:**
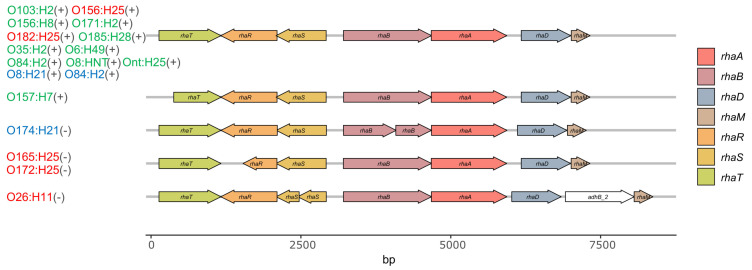
Gene map of the *rha* operon in bovine STEC. Genes relevant for the uptake of rhamnose encode for the rhamnose/proton-symporter RhaT in the inner membrane of *E. coli* and the transcriptional activators RhaS and RhaR [29]. Genes involved in the metabolism of rhamnose encode the rhamnulose kinase RhaB, the rhamnose isomerase RhaA, the rhamnulose-1-phosphate aldolase RhaD [31], and RhaM, a rhamnose mutarotase [30,32]. Serotypes in red represent STEC^per^, in green STEC^spo^, in blue STEC with unknown colonization type; the respective L-rhamnose utilizing phenotype is shown in parentheses (−, negative; +, positive). Strains included in this study and belonging to the same serotype did not differ in their *rha* operon and are therefore not depicted individually in the figure.

Analysis of the *glc* operon, which is involved in glycolate utilization, shows that *glcA*, encoding the permease for glycolate uptake [27], was missing (O26, O157), disrupted (O182) or truncated by a frameshift (O165, O172) in nearly all STEC strains deficient in glycolate-, glyoxylate- or tartrate-dependent metabolism (Figure 6). Despite their inability to grow on glyoxylic acid, the O156-STEC^per^ strains possess a complete *glc* operon, while, in contrast, no O157 STEC^spo^ possess a *glc* operon, but were positive for glyoxylic acid metabolism. Of note, all O26 STEC^per^ strains were indistinguishable in the genetic organization of the operon, although two strains were glycolic and glyoxylic acid positive in the Omnilog^®^ assay and two strains were not (Figure 7).

We also assessed the Illumina sequences of the STEC strains for the presence of *rhaT*, the gene encoding the rhamnose/proton symporter [28], responsible for rhamnose uptake, *rhaS/rhaR*, the transcriptional activators of *rhaT* expression [29], as well as the genes *rhaB*, *rhaA*, *rhaD*, and *rhaM* encoding proteins involved in rhamnose catabolism [30]. This revealed that the deficiency of O26 STEC^per^ to utilize rhamnose correlates with a frameshift in *rhaS* and that of O165/O172 STEC^per^ with a frameshift in *rhaR*, both resulting in truncated transcriptional activator proteins. All four O157 STEC^spo^ strains had a frameshift in the *rhaT* gene, resulting in an N-terminally truncated symporter protein, but tested positive in the phenotype microarray for rhamnose utilization.

## 3. Discussion

Cattle are the most relevant source of human EHEC infections, and the majority of cattle are believed to harbor STEC strains. Evidence exists that, under farming conditions, shedding and transmission dynamics differ between STEC strains in the bovine reservoir [33,34,35], yet the molecular basis for certain colonization and shedding patterns is only recently being unveiled [7]. Longitudinal studies examining fecal shedding of STEC by the bovine reservoir host allowed us to distinguish two different groups of STEC: strains isolated either over several weeks or months and therefore designated as persistent colonizing STEC strains (STEC^per^) or STEC strains excreted only for short time periods, referred to as sporadic colonizers (STEC^spo^) [22]. Subsequent studies failed to link distinct genetic markers with the colonization type of the strains [26]. Moreover, the genetic distance between the different geno-serotype groups of STEC^per^ strains [36] was greater to each other than to the STEC^spo^ strains, indicating a more distinct and separate evolution. In order to disclose commonalities of strains exhibiting one of either colonization habits, phenotypic differences between the persisting and sporadic colonizing strains were looked for in the present study.

Some STEC strains possess a very low infectious dose of less than 100 bacteria in humans [37]. One factor contributing to such a low infectious dose might be an enhanced acid tolerance that enables the pathogen to survive the passage through the human stomach without gross damage. In the adult cow’s four forestomachs, only the abomasum exhibits an acidic milieu with pH 2–2.5 in its lumen [38,39], similar to the pH value of 1.5–2 in the human monogastric stomach [40]. Based on this, it is tempting to assume that strains traversing more frequently through the stomach or abomasum, i.e., STEC^spo^ strains, exhibit a higher acid tolerance than persisting strains. In *E. coli*, several acid resistance systems (AR) have been described: one oxidative system that is repressed by a yet unknown glucose metabolite and regulated by RpoS (named AR1) and systems based on the decarboxylation of amino acids (the glutamate [AR2 or GDAR], the arginine [AR3, ADAR], the lysine [AR4, LDAR] or the ornithine system [ODAR]) [41]. The assay setup used here (LB broth, aerobic incubation) primarily monitors the functionality of the AR1 system [42], which is the AR system that provides the bacteria with the highest level of protection, functioning at pH 2 or even less [43]. Accordingly, STEC strains analyzed in this study with a truncation or complete deletion of the *rpoS* gene displayed either no or only reduced acid resistance. Overall, testing revealed no clear correlation between colonization type and acid resistance of the strains, since the STEC^per^ group comprised both, strains with the highest and the lowest acid tolerance. However, the *rpoS* gene is probably non-functional in five out of eleven STEC^per^ tested (45.5%), but only in three out of 14 STEC^spo^ strains (21.4%). This supports our hypothesis that the potential of the strains to adjust to milieu changes via the global regulator RpoS that regulates acid resistance, among other stress conditions, might be more important in STEC^spo^ than in STEC^per^. Mutations in *rpoS* are common in *E. coli* and STEC isolates and are assumed to reflect an increased ability to scavenge for scarce nutrients at the expense of stress protection (see [44] and references cited therein). Analysis of O157:H7 isolates related to a spinach-associated outbreak revealed the presence of *rpoS* mutations in clinical isolates as opposed to the wild-type allele found in strains from environmental sources. The niche associated with the selection for the mutants was not identified [44].

Strains with a shorter retention time in the host must, in turn, have strategies to survive in the environment, while strains persisting in the gut need to adapt to this niche. Biofilms can serve to protect the *E. coli* strains from adverse conditions either within the host or outside in the environment. Biofilm formation by *E. coli* mainly relies on the production of cellulose (encoded in the *bcs* gene cluster) and curli (encoded by the *csg* genes) [45,46]. Expression of both requires CsgD, a transcriptional regulator directly inducing the *csgBAC* operon and indirectly inducing cellulose production by enhanced *adrA* transcription [47]. The signal cascade to regulate *csgD* additionally includes the *rpoS*-encoded sigma factor as well as MlrA [48,49]. To monitor host- versus environment-adapted conditions, we varied the incubation temperature from 20 °C (approximated environmental conditions) to 37 °C (approximated conditions inside the host). The STEC^spo^ strains seemed to be better adapted to environmental conditions as they produced significantly more biofilm at 20 °C than the STEC^per^ strains. Interestingly, STEC^per^ produced nearly no biofilm at 20 °C. These results are in line with results showing that plant-associated *E. coli* produce significantly more biofilm and curli at lower temperatures than host-associated isolates from the ECOR collection [50]. To identify the genetic background for this phenotype, we assessed several genes related to biofilm production (*bcs* genes, *csg* genes, *adrA*, *rpoS*, *mlrA*) in the WGS data. Cellulose- and curli-encoding gene clusters (*bcs* and *csg*) were present in all strains tested. Besides differences in the *rpoS* gene as mentioned above, the integrity and presence of the *mlrA* gene differed between the STEC strains. In the STEC O157:H7 strains, the insertion of the *stx1*-carrying prophage often disrupts the *mlrA* (synonymous *yehV*) gene [51]. The truncated MlrA protein is then unable to up-regulate transcription of *csgD*, the central regulator of biofilm formation [52]. Besides the O157 STEC^spo^ strains, we found the majority of the STEC^per^ strains to possess an only 215-residue-long truncated MlrA, and we could verify in PacBio sequences, additionally available for some strains under study here [53], that the disruption was due to the insertion of an *stx1*-carrying prophage.

Such strains were similarly unable to produce biofilm in greater amounts, suggesting that biofilm production might not be as relevant for persistently colonizing STEC as for strains more likely to be exposed to environmental conditions.

Depending on the preferred niche(s) of sporadic- and persistent-colonizing strains, their metabolic abilities might differ. We checked this with the Omnilog^®^ system that allows measuring the utilization of single carbon, nitrogen, sulphur and phosphorus substrates by quantification of the respective strain’s respiratory activity. From a total set of 379 different substrates available as a sole nutrient, STEC^per^ and STEC^spo^ utilized only 15 substrates (8 C- and 7 S-sources) to significantly different extents. Using bioinformatics, a cascade of two key substrates, glyoxylic acid and L-rhamnose, allowed for differentiating STEC^per^ from STEC^spo^. The importance of glyoxylate metabolism in the lifestyle of STEC^spo^ is substantiated by the presence of the three components glyoxylic acid, glycolic acid, and m-tartaric acid as substrates that were differently utilized by STEC^per^ and STEC^spo^.

To understand why these substrates were differently utilized, WGS data of the strains were searched for the presence and integrity of genes involved in glyoxylate metabolism. This particularly included the *glc*-operon that comprises seven genes. All events leading to gene inactivation were associated with *glcA*, the gene encoding the glycolate permease responsible for glycolate uptake [27]. Differing between serotypes, three types of *glcA* inactivation were identified: two were insertions of a mobile element (*traT* in O26 and *IS*3 in O182 STEC^per^ strains) and one the truncation of the open reading frame due to a frame shift (in O165/O172 STEC^per^ strains). All other genes in the cluster were found to be intact, implying that the bacteria can use the glyoxylate cycle as an anabolic pathway for the synthesis of carbohydrates during regular metabolism when glycolic acid, glyoxylic acid or m-tartaric acid are synthesized by other pathways. The loss of GlcA in STEC^per^ may be fostered by the fact that glycolate is produced in plants as an intermediate of photorespiration when oxygen is available but glycolate may not be readily available in the ingesta in the bovine gut due to the low partial pressure of oxygen. In contrast, STEC^spo^, which might be more frequently in contact with metabolically active plants in the environment, would have a clear benefit by retaining the ability to acquire and metabolize glycolate as an additional C-source.

By contrast, inspection of the *rha* operon revealed that the missing metabolic activity does not only result from the alteration of just a single gene, but that several different genes have rather lost their functionality by independent mutation events. The *rha* operon comprises seven genes, one of which encodes RhaT, the rhamnose/proton-symporter in the inner membrane. Two genes encode the transcriptional activators RhaS and RhaR [29]. Additionally, genes encoding proteins involved in the metabolism of rhamnose, e.g., the rhamnulose kinase RhaB, the rhamnose isomerase RhaA, the rhamnulose-1-phosphate aldolase RhaD [31], and RhaM, a rhamnose mutarotase, are present [30,32]. Only three of these seven genes, *rhaA*, *rhaD*, and *rhaM*, seem to encode full-length proteins in all strains, although the presence of amino acid substitutions in these three genes in any of the strains hampers any prediction of gene functionality. In contrast, in strains not able to metabolize rhamnose, the open reading frames of the *rhaB, rhaS* or *rhaR* genes are obviously dysfunctional. These three genes are truncated or split into two putative protein-encoding fragments by frameshifts as the result of single nucleotide deletions (*rhaB, rhaR*) or insertions (*rhaS*). It is surprising that the O157 STEC^spo^ strains are able to utilize L-rhamnose because the predicted RhaT seems to be truncated. According to Tate and Henderson [54], the RhaT symporter consists of 10 transmembrane helices. In O157 STEC^spo^, the first two helices are missing. We assume that the symporter is still functional, as another L-rhamnose transporter has not been described in *E. coli* to date and the genes encoding RhiTN, a protein complex described to transport rhamnose-containing oligosaccharides [30], were not present in our strain collection.

The missing correlation between pheno- and genotype in some strains lacks an obvious explanation. The O26 STEC^per^ strains all possess the same disruption of *glcA*, but two out of the four strains were able to grow when only glycolic or glyoxylic acid was available. Furthermore, O156 STEC^per^ strains possess a full-length *glcA* without any amino acid substitution compared to most positive STEC^spo^ strains but are not able to metabolize glycolic or glyoxylic acid. Similarly, three O165/O172 STEC^per^ strains utilized a significantly lower number of substrates than the fourth O165 STEC^per^ strain and all other STEC strains assessed, but did not possess any obvious genome differences to the other strains of the O165/O172 STEC^per^ group. Such discrepancies have also been observed in other *E. coli* WGS/phenotype analyses. Alqasim, et al. [55] showed that *E. coli* strains of MLST ST131 exhibited heterogeneous metabolic phenotypes, and, obviously, did not belong to one metabolically distinct lineage. This underlines the difficulties in predicting exerted phenotypes solely based on sequence data. This problem is also well known when trying to deduce susceptibilities of strains against antimicrobials based on molecular genetic analyses [56,57]. In order to mechanistically understand the metabolic backgrounds of the strains, a deeper analysis of the genome data taking into account the frequently complex interactions decisive for a specific phenotype is clearly necessary.

It is noteworthy that several different inactivation events affected the integrity of glyoxylic acid uptake or rhamnose metabolism in STEC^per^ strains. Including previously published genome analyses of the strains, it has to be considered that the convergent development of the different STEC^per^ strain clusters by gaining similar virulence-associated gene patterns and the inactivation of single metabolic pathways are not independent evolutionary events [26]. One such example is the disruption of *mlrA* by *stx1*-converting bacteriophages. It was recently shown that carriage of the *stx*-converting phage profoundly affects *E. coli* gene expression and carbon source utilization [58]. Phage-encoded regulators may mediate such effects, but prophage-encoded metabolic genes such as *nanS-p* also have an impact on the overall metabolic capabilities of the affected strains [59]. Due to the loss of *stx*-converting prophages in some strains, we were able to investigate the influence of the prophages on the metabolic profiles of very similar strains, but contrary to the published data, we did not find significant differences (data not shown). We therefore believe that the differences in metabolic traits described herein are not related to the presence or absence of prophage sequences, and the conclusion that these differences have impacted the colonization pattern of the STEC parent strains in the cattle population sampled is valid. Nevertheless, we cannot exclude that gene products of single prophages might modulate the gene expression in STEC, but a global effect across different phage types could not be confirmed.

It will be interesting to determine if the key substrates found in this limited set of strains from a single region in Germany also allow the discrimination between STEC^per^ and STEC^spo^ in larger collections of strains and in other geographic regions. Basing the decision tree on a broader combination of pheno- and genotypic markers is likely to help improve the predictive value of this approach. Despite these limitations, the current study was able to correlate bovine STEC colonization types to distinct in vitro phenotypes and, for the most part, associate these phenotypes with changes in the genetic backgrounds. The loss of certain phenotypes in STEC^per^ is in line with their presumed stronger host association compared to more generalist strains [60]. The proposed metabolic and genetic distinction between persistent and sporadic colonizing strains helps us to better understand host and niche adaptation of *E. coli* strains. In a human outbreak scenario, identifying the causative agent as “bovine-persistent” STEC might indicate a meat-associated origin, while identification of “sporadic” STEC might hint at an environmental contribution. Classification of STEC strains is also instrumental for developing novel approaches to tackle STEC^per^ strains which serve as the genetic source for the continuous generation of novel STEC strains in the reservoir host [61].

## 4. Material and Methods

### 4.1. Strain Collection

From a collection of 178 whole genome sequenced and genotypically characterized Shiga toxin-producing *Escherichia coli* (STEC) [26], 28 strains were selected according to their MLST and virulence-associated gene patterns to represent the genomic diversity of this STEC population. All strains were originally isolated based on the presence of an *stx* gene [22,35], but several strains had lost the bacteriophage-encoded genes during storage as described previously [26,62]. Nevertheless, all strains were designated “STEC” in the current study. Relevant characteristics of all strains investigated in the current study are listed in Table 2.

Additionally, two apathogenic *E. coli* strains served as controls in several assays: *E. coli* Nissle 1917 (EcN; kindly provided by PD Dr. U. Methner, Jena) and *E. coli* K-12 (C600; kindly provided by Prof. Dr. R. Bauerfeind, Gießen).

### 4.2. Illumina Sequencing

The strains were whole genome sequenced using Illumina MiSeq (Illumina Solutions Center, Berlin, Germany) 300 bp paired-end sequencing with greater than 40× coverage. The sequence read data were first subjected to quality control using the NGS tool kit [64]. Reads with a minimum of 70% of the bases having a phred score of greater than 20 were defined as high quality reads. De novo assembly of the resulting high-quality filtered reads into contiguous sequences (contigs and scaffolds) was conducted using the SpaDes assembler in careful mode to enable additional mismatch correction. All contigs of length longer than 500 bp were included in the final assembly. Annotation of coding sequences was conducted with Prokka (default settings). Raw NGS files (fastq) were uploaded to the NCBI SRA database (Bioproject no. PRJNA559322).

### 4.3. Genome Analysis

To check the presence or absence of specific genes within the WGS scaffolds, the command line tool blastp was used. Reference protein sequences from the *E. coli* K-12 strain MG16255 were aligned to translated and annotated coding sequences of our genome assemblies (percentage identity >40% and query coverage >50%) to identify the location of the genes. To visualize the composition and similarity of the gene clusters identified this way, we used the R packages rtracklayer, Biostrings, and gggenes. They provided helpful functions to load, process, and plot the structure of gene clusters in our strains. Further analyses at the nucleotide level were conducted by mapping the contig sequences to the reference sequence (Geneious version 8.1.3; Biomatters Ltd., Auckland, New Zealand).

### 4.4. Acid Resistance Assay

Survival rates of the bacteria in media with different pH values were measured by a protocol modified from Coldewey, et al. [65]. Bacteria (7.3 ± 0.2 log10 cfu) from an overnight culture in LB broth (37 °C, 180 rpm) were inoculated in 1 mL fresh LB broth with the pH adjusted to 1.5, 2.5 or 7.8 (acidified with HCl) and incubated (2 h, 37 °C, without shaking). Colony forming units (cfu) were calculated from the overnight culture to confirm the inoculation dose as well as from the inoculated broth cultures after the 2 h incubation. For this, log10 serial dilutions were prepared in phosphate-buffered saline (PBS), and one aliquot of 100 µL from each of the first two dilution steps and two aliquots of 10 µL from each further dilution were spotted on sheep-blood agar plates. After incubation (overnight, 37 °C), single colonies were counted in the dilution steps which contained 10–100 colonies, and the cfu/mL was calculated. The acid resistance assay was performed with three biological replicates for each strain.

### 4.5. Biofilm Crystal Violet Plate Assay

Biofilm production was quantified by a crystal violet assay modified from O’Toole [66]. In brief, bacteria from an overnight culture in LB broth (37 °C, 180 rpm) were diluted 1:100 in salt-free lysogeny broth (LBnoS broth) and plated in quadruplicate in 96-well polystyrene microtiter plates (150 µL/well). After incubation (24–48 h, 20 °C, 28 °C or 37 °C), the liquid cultures were removed and the wells rinsed with dH_2_O. The biofilm was stained with 0.1% crystal violet solution (15 min) and then the wells washed three times with dH_2_O. After drying, 200 µL 20% acetic acid/well was added and incubated (15 min, room temperature). Finally, 100 µL were transferred to a new plate and the OD_590 nm_ measured. Four wells, containing LBnoS broth without bacteria, served as a negative control.

### 4.6. Curli Detection by Congo Red Agar

LBnoS agar plates containing 40 µg/mL Congo red dye and 20 µg/mL Coomassie Brilliant Blue G were spotted with 5 µL LB of an overnight culture of an STEC strain and incubated for up to 48 h at 20 °C, 28 °C or 37 °C [67]. Colonies were macroscopically checked for color and consistency of the surface. Strains expressing curli are able to bind the Congo red dye and are stained brown, strains producing cellulose pink, and strains producing both exhibit the rdar phenotype (red, dry, and rough).

### 4.7. Metabolic Phenotype Microarray (Omnilog^®^)

The metabolic activity of the strains was measured using the Omnilog^®^ phenotype microarray (PM) system (Biolog Inc., Hayward, CA, USA). At least three biological replicates of each strain were tested in a microtiter plate-based microarray for the metabolism of 190 different carbon (plates PM1 and PM2A), 95 nitrogen (plate PM3B), 59 phosphorus, and 35 sulfur (both plate PM4A) sources. For the assay, the strains were freshly grown on sheep-blood-agar (Sifin, Berlin, Germany; overnight, 37 °C). The PM plates were inoculated with a bacterial suspension containing approx. 10^5^ cells/100 µL/well prepared in IF-Oa medium containing Dye-Mix A and, in the case of plates PM3B and PM4A, 20 mM Na-succinate as recommended by the manufacturer. Plates were incubated (48 h, 37 °C) and bacterial respiration measured every 15 min by reduction of the tetrazolium violet dye.

### 4.8. M9 Minimal Medium Growth Kinetics with Differing C-Sources

The utilization of selected carbon sources (L-rhamnose and glyoxylic acid [Sigma–Aldrich Chemie GmbH, Munich, Germany]) was additionally tested by measuring the growth kinetics of the strains in M9 medium (M9 Minimal Salts; Fisher Scientific GmbH, Nidderau, Germany) supplemented with 2 mM MgSO_4_, 0.1 mM CaCl_2_, the respective carbon source (0.4%), and, if necessary, the pH adjusted to pH 7. Then, LB overnight cultures of the STEC strains were diluted 1:100 in 800 µL in M9 medium and plated in 48-well microtiter plates. The plates were incubated (24 h, 37 °C) in an Epoch2T reader (BioTek Instruments GmbH, Bad Friedrichshall, Germany) with continuous linear shaking and the OD_600 nm_ measured every hour. The control was one well per strain with M9 medium without any carbon source on each plate. Each strain was tested with each carbon source in three biological replicates.

### 4.9. Data Analysis

The metabolic activity was analyzed with the opm package in the statistical environment R [68,69]. Kinetic data of the Omnilog^®^ units were exported as Microsoft^®^ Excel files and loaded with the function “read_opm”. The area under the curve (AUC) was calculated with the opm_fast method for the first 48 h of the growth kinetics with one measurement per hour. After subtraction of the negative control (plate- and substrate-specific), the values were rated as follows: <500 negative and ≥500 positive. Substrates with constant positive or negative levels in most strains were discarded, i.e., only substrates with at least three positive or three negative samples were selected for further analysis. The cutoff value of 500 was approximated from histograms of all plate distributions. Significant differences in the mean AUC of grouped strains (persistent and sporadic) were calculated using a two-sample Wilcoxon test and a *p*-value cutoff of 0.05. All further analyses were based on a preprocessed dataset, which included only eight C-substrates with significantly different utilization between the colonization groups.

For substrate multidimensional data analysis, we performed a series of visualization, classification, and selection methods. First, a two-dimensional scatterplot of the dataset (eight substrates, 25 strains) was generated by using a scaling (cmdscale) approach. The resulting plot allowed us to visually inspect the cluster structure of the data. Prior to classification, we decided to transform our dataset into a binary format (0 = negative, 1 = positive) to facilitate the classification process and remove unnecessary bias from the data. A binary heatmap was generated with function pheatmap, which orders rows (strains) and columns (substrates), applying a hierarchical clustering (average linkage). Next, random forest classification was conducted using the colonization type (persistent, sporadic) as a class attribute. Here, we applied the packages caret and randomForest. Identification of substrates that allowed for predictions of the colonization type was the main objective. We performed repeated cross-validation (10-fold, 50-repeats) on the dataset and evaluated the predicted classes in a receiver operating characteristic (ROC) analysis. AUC ROC values and confidence intervals were computed with the pROC package to quantify the performance of both sensitivity and specificity and to compare them between different substrate sets. To rank substances according to their effect on classification, we analyzed the random forest importance values (mean decrease in accuracy). Those performance values provide estimates about the importance of each substrate and were used in a recursive feature elimination to optimize the classifier, which was implemented by the rfe function in the caret package. A final representative decision tree was learned with the rpart package to visualize the tree structure and resulting decision paths.

Differences in the potential of the strains to produce biofilms were analyzed by comparison of the mean OD_590 nm_ of four biological replicates by a Dunnett-T test to be significantly greater than the mean of the negative control *E. coli* K-12 strain at the respective incubation temperature.

## Figures and Tables

**Table 1:** Survival of Shiga toxin-producing *Escherichia coli* (STEC) strains after 2 h incubation in acidic lysogeny broth (LB broth, pH 1.5 and pH 2.5), measured by colony forming units (cfu) after 2 h incubation in LB broth adjusted to the respective pH value and shown as mean log10 cfu/mL of three biological replicates.

Strain	Geno-Serotype	log10 cfu/mL (Mean of n = 3)				log10 Reduction Compared to Control pH 7.8		RpoS Protein
		Inoculum	pH 1.5	pH 2.5	pH 7.8	pH 1.5	pH 2.5	
Persistent Colonization Type (STEC^per^):								
13E0591	O26:H11	7.33	0.52	7.02	8.23	7.71	1.21	full-length
13E0663	O26:H11	7.31	1.00	6.89	7.83	6.83	0.95	full-length
13E0674	O26:H11	7.42	3.91	6.96	7.93	4.02	0.97	full-length
13E0634	O26:H11	7.36	3.43	6.88	7.95	4.52	1.07	full-length
13E0753	O156:H25	7.46	n.d.	5.10	8.29	8.29	3.19	full-length
13E0780	O156:H25	7.58	n.d.	3.35	8.24	8.24	4.89	truncated (26 AS) due to an SNP at pos. 79_G__→__T_
13E0725	O182:H25	7.43	n.d.	3.17	8.27	8.27	5.10	truncated (289 AS) due to a frame shift at pos. 847 (deletion of 4 nucleotides)
13E0711	O165:H25	7.26	0.82	5.51	7.77	6.94	2.25	full-length
13E0718	O165:H25	7.47	n.d.	5.76	7.90	7.90	2.14	truncated (177 AS) due to an SNP at pos. 559_C__→__G_
13E0734	O165:H25	6.99	2.90	4.51	7.40	4.50	2.90	deleted
13E0812	O172:H25	7.30	n.d.	1.82	8.03	8.03	6.20	truncated (189 AS) due to an insertion of the phage-related gene *mom*
Sporadic Colonization Type (STEC^spo^):								
13E0609	O157:H7	7.32	n.d.	2.62	7.70	7.70	5.08	deleted
13E0613	O157:H7	6.99	1.82	5.14	7.52	5.70	2.39	truncated (70 AS) due to a 22 nucleotide insertion at pos. 170
13E0762	O157:H7	7.16	n.d.	4.43	7.38	7.38	2.95	full-length
13E0793	O157:H7	6.99	n.d.	3.97	7.78	7.78	3.80	full-length
13E0611	O84:H2	7.51	n.d.	5.74	7.91	7.91	2.17	full-length
13E0822	O103:H2	7.45	2.43	5.66	8.37	5.94	2.70	full-length
13E0704	O185:H28	7.48	4.53	7.16	8.37	3.84	1.20	full-length
13E0895	O8:H_NT_	7.48	1.30	5.49	8.32	7.01	2.82	deleted
13E0899	Ont:H25	7.39	n.d.	2.52	8.39	8.39	5.87	331 AS, due to a duplication of triplet GTA at pos. 307
13E0659	O35:H2	7.51	5.00	7.14	8.20	3.20	1.06	full-length
13E0867	O171:H2	7.36	0.52	5.35	8.22	7.69	2.87	full-length
13E0869	O6:H49	7.44	2.32	5.60	8.05	5.74	2.45	full-length
13E0892	O6:H49	7.23	1.60	6.17	7.98	6.37	1.81	full-length
13E0767	O156:H8	7.57	n.d.	6.75	8.30	8.30	1.55	full-length
Unknown Colonization Type:								
12E0115	O84:H2	7.41	6.26	7.37	7.88	1.62	0.52	full-length
12E0117	O8:H21	7.52	2.23	7.22	7.82	5.59	0.60	full-length
12E0119	O174:H21	7.13	n.d.	6.97	8.27	8.27	1.30	deleted
Control Strains:								
EcN	O6:K5:H1	7.40	n.d.	3.70	8.32	8.32	4.63	not known
C600	K-12	6.75	1.94	3.76	8.27	6.33	4.50	not known

EcN, *E. coli* Nissle 1917; n.d. = not detectable; RpoS protein full length = *rpoS* gene encoding a 330 amino acid long RpoS protein, positions refer to the gene; SNP = single nucleotide polymorphism.

**Table 2:** *E. coli* strain collection used in this study.

Strain	Geno-Serotype	MLST ^1^		Phylo-Group ^2^	Selected VAGs ^3^			SRA ^4^ Accession No.
		ST Complex	ST		*stx* Subtype	Other Toxins	Adhesins	
Strains Representing the Persistent Colonization Type (STEC^per^):								
13E0591	O26:H11	ST29 Cplx	21	B1	none	*astA, ehxA, toxB*	*β-eae, efa-1, iha*	SRR9972680
13E0663	O26:H11	ST29 Cplx	21	B1	1a	*astA, ehxA*	*β-eae, efa-1, iha*	SRR9972674
13E0674	O26:H11	ST29 Cplx	21	B1	1a, 2a	*astA, ehxA, toxB*	*β-eae, efa-1, iha*	SRR9972669
13E0634	O26:H11	ST29 Cplx	1705	B1	1a, 2a	*astA, ehxA, toxB*	*β-eae, efa-1, iha*	SRR9972676
13E0753	O156:H25	none	688	B1	1a	*astA, ehxA*	*ζ-eae*	SRR9972663
13E0780	O156:H25	none	300	B1	1a	*astA, ehxA*	*ζ-eae*	SRR9972672
13E0725	O182:H25	none	300	B1	1a	*astA, ehxA*	*ζ-eae*	SRR9972665
13E0711	O165:H25	none	119	A	none	none	*ε-eae*	SRR9972667
13E0718	O165:H25	none	119	A	2a	*astA*	*ε-eae, efa-1*	SRR9972668
13E0734	O165:H25	none	119	A	2a	*astA*	*ε-eae, efa-1*	SRR9972666
13E0812	O172:H25	none	660	A	2a	*astA*	*ε-eae, efa-1*	SRR9972661
Strains Representing the Sporadic Colonization Type (STEC^spo^):								
13E0609	O157:H7	ST11 Cplx	11	D	2c	*astA, cdt, ehxA, toxB*	*γ-eae, iha*	SRR9972677
13E0613	O157:H7	ST11 Cplx	11	D	1a, 2c	*astA, cdt, ehxA, toxB*	*γ-eae*	SRR9972675
13E0762	O157:H7	ST11 Cplx	11	D	none	*astA, cdt, ehxA, toxB*	*γ-eae, iha*	SRR9972664
13E0793	O157:H7	ST11 Cplx	11	D	none	*astA, cdt, ehxA, toxB*	*γ-eae, iha*	SRR9972662
13E0611	O84:H2	none	306	B1	1a	*astA, cdt, ehxA*	*ζ-eae*	SRR9972678
13E0822	O103:H2	ST20 Cplx	17	B1	1a	*ehxA*	*ε-eae, efa-1*	SRR9972660
13E0704	O185:H28	none	658	D	2a	*ehxA*	LEE-neg., *iha, saa*	SRR9972670
13E0895	O8:HNT	ST155 Cplx	155	D	none	none	LEE-neg.	SRR9972656
13E0899	Ont:H25	ST155 Cplx	58	B1	2a	*astA, ehxA, subA*	LEE-neg., *iha, saa*	SRR9972655
13E0659	O35:H2	none	5266	B1	none	none	*β-eae*	SRR9972673
13E0867	O171:H2	none	332	B1	2d	none	LEE-neg., *iha*	SRR9972659
13E0869	O6:H49	none	1079	B1	none	none	LEE-neg., *iha*	SRR9972658
13E0892	O6:H49	none	1079	B1	1a	none	LEE-neg., *iha*	SRR9972657
13E0767	O156:H8	none	327	B1	none	none	*θ-eae, iha*	SRR9972671
Strains with Unknown Colonization Type:								
12E0115	O84:H2	none	306	B1	1a	*astA, ehxA*	*ζ-eae*	SRR9972681
12E0117	O8:H21	none	1794	B1	2d	*astA*	LEE-neg.	SRR9972682
12E0119	O174:H21	none	677	B1	2c	none	LEE-neg.	SRR9972679

^1^ MLST, multi locus sequence typing; ^2^ PCR according to Clermont, et al. [63]; ^3^ VAGs (virulence associated genes) as described by Barth and colleagues [26]; SRA, Sequence Read Archive of the NCBI, Bethesda, MD, USA.

## Supplementary Materials

The following are available online at https://www.mdpi.com/2072-6651/12/6/414/s1, Figure S1: Metabolic activity of each strain on the Omnilog® plates tested (PM1, PM2A, PM3B, PM4A), shown as AUC (area under the curve) after subtraction of the respective negative control. Each AUC is the mean of three biological replicates.; Figure S2: S-Substrates with significantly different metabolic activity in STEC^per^ and STEC^spo^. Shown are the AUC values of each group as Box Whisker plots of the discriminatory sulfate substrates with significant (*p* < 0.05) differences in the mean AUC between STEC^per^ (red) and STEC^spo^ (green). The STEC strains with an unknown colonization type are depicted in blue; Figure S3: Correlation of two test systems to quantify the utilization of glyoxylic acid or L-rhamnose as single C-source by bovine STEC as measured by respiration (Omnilog^®^) and growth (M9). For correlation determination, the AUC values were calculated for three replicates of each strain during a 24 h incubation period. Glyoxylic acid (black dots) correlates with r = 0.822 (y = 0.1547x − 385.47) and L-rhamnose (grey triangles) with r = 0.802 (y = 0.2475x − 267.77). Table S1: Raw data of the metabolic activity of each strain on the Omnilog^®^ plates tested (PM1, PM2A, PM3B, PM4A) as mean AUC after 48 h incubation at 37 °C of three biological replicates.
